# Identification of a LolB-like protein in *Porphyromonas gingivalis* reveals selective LolA–LolB pairing

**DOI:** 10.1038/s41598-026-49975-1

**Published:** 2026-04-22

**Authors:** Deepika Jaiman, Makoto Hirohata, Yoshiaki Hasegawa, Karina Persson

**Affiliations:** 1 Centre for Microbial Research (UCMR), Umeå University, Umeå, Sweden; 2 Department of Chemistry, Umeå University, Umeå, Sweden; 3 Department of Microbiology, School of Dentistry, Aichi Gakuin University, Nagoya, Japan

**Keywords:** Lipoprotein transport, LolB, Crystal structure, Bacteroidota, Biochemistry, Microbiology, Molecular biology

## Abstract

The lipoprotein transport (Lol) system is essential for outer membrane biogenesis in Gram-negative bacteria, yet its composition and organization vary markedly across bacterial phyla. While lipoprotein transport via the Lol pathway has been extensively characterized in *Escherichia coli*, its components in the Bacteroidota phylum remain poorly understood. *Porphyromonas gingivalis*, a major periodontal pathogen has long been thought to lack the outer membrane lipoprotein insertase LolB, leaving the mechanism of lipoprotein insertion unclear. Here, we have identified and characterized a LolB-like protein in *P. gingivalis* (LolB-PG). We determined its crystal structure at 2.1 Å resolution and revealed a conserved LolB fold but with an enlarged and more accessible lipid-binding cleft compared to proteobacterial homologs. Biophysical analyses demonstrate that LolB-PG selectively interacts with the cognate periplasmic chaperone LolA but not with the paralog LolA3, indicating a conserved yet specific LolA–LolB pairing. Deletion of the gene encoding LolB*-*PG did not affect bacterial growth or the assembly, localization, or formation of type-V fimbriae—which are polymerized from lipoproteins— suggesting the existence of alternative lipoprotein trafficking routes in *P. gingivalis*. Together, our findings reveal that Bacteroidota encode a functional LolB-like protein and highlight diversification of lipoprotein transport pathways beyond well-studied γ-proteobacteria.

**Supplementary Information:**

The online version contains supplementary material available at 10.1038/s41598-026-49975-1.

## Introduction

Gram-negative bacteria are protected by a complex cell envelope consisting of an inner membrane (IM), an outer membrane (OM), separated by the peptidoglycan containing periplasm. The IM is largely made up of a symmetric phospholipid bilayer containing integral membrane proteins that consist mainly of transmembrane helices. The OM is an asymmetric lipid bilayer where the inner leaflet is built up from phospholipids and the outer leaflet is decorated with lipopolysaccharides (LPS) that provide stability and protection against external threats^1^. The proteins that traverse the OM are mainly β-barrel proteins, also known as porins, that selectively can transfer molecules and ions across the membrane. In addition, numerous lipoproteins are anchored to both membranes. These proteins are synthesized in the cytoplasm as precursors and undergo stepwise modifications to acquire three N-terminal lipid anchors that tether them to the membranes—a process essential for bacterial viability. In the precursor proteins a cysteine residue immediately following the signal peptide serves as the site of acylation, and its first acylation is carried out by the enzyme lipoprotein diacyl glyceryl transferase (Lgt) that adds two acyl chains to the cysteine sulfhydryl. Next, the signal peptide is removed by the signal peptidase LspA which generates a free amino group on the diacylated cysteine. Finally an acyl group is added to this amino group by the enzyme apolipoprotein N-acyltransferase (Lnt), resulting in a triacylated protein^2^.

**Fig. 1:**
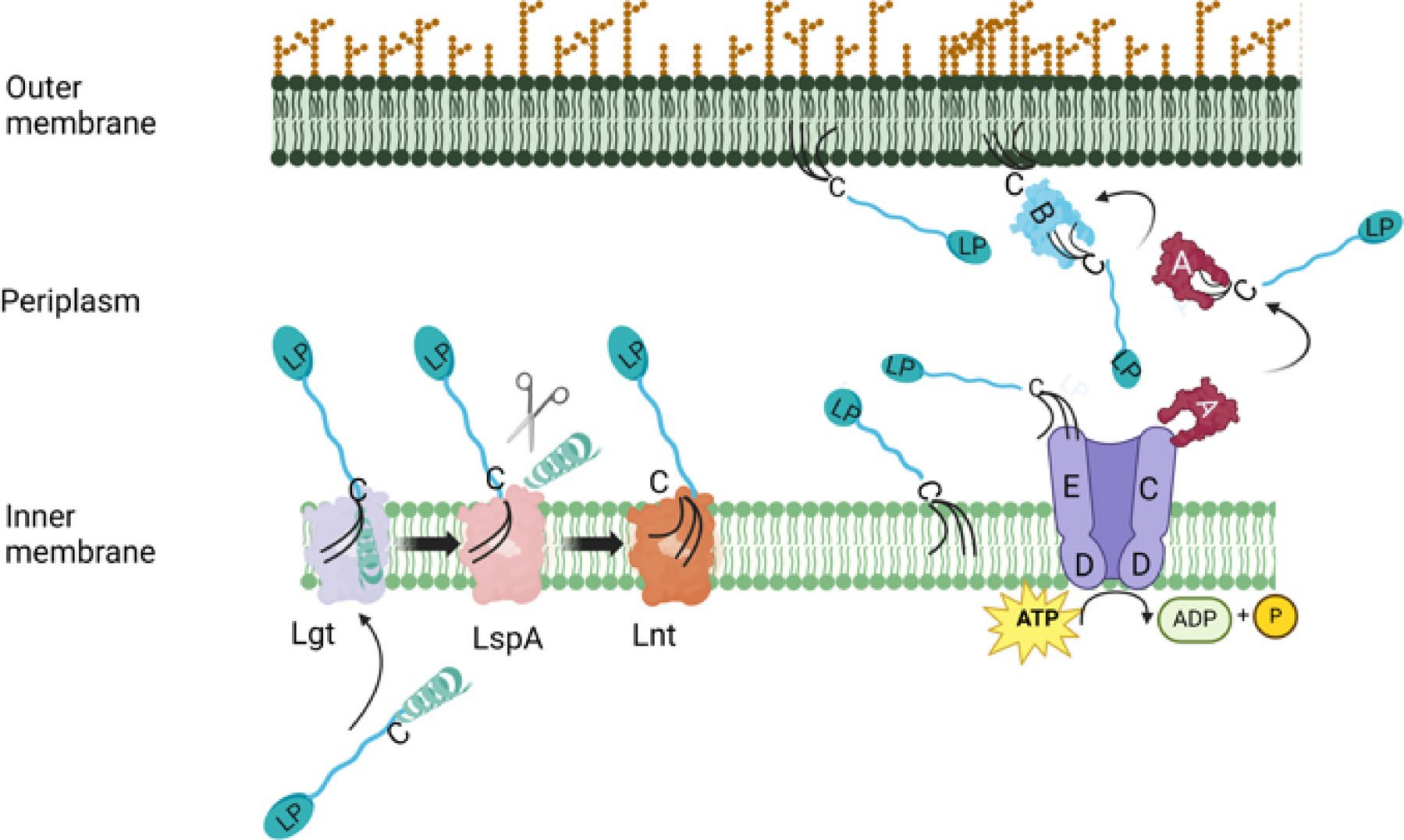
Overview of the acylation and lipoprotein transport pathway. The prelipoprotein is transported over the IM where it is modified: two acyl chains are added to the side chain of a conserved cysteine by Lgt, the signal peptide is removed by LspA and the enzyme Lnt adds a third acyl chain to the amino group of the diacylated cysteine. If the mature lipoprotein (LP) is destined to the OM the LolCDE complex will extract it from the membrane and transfer it to the chaperone protein LolA. The lipoprotein is delivered to LolB, which will relocate it to the OM. This scheme is based on the *E. coli* system. LolB has previously only been reported in β- and γ- proteobacteria and not the phylum bacteroidota. The figure was created with BioRender.com.

For lipoproteins destined for the OM, transport across the periplasm is mediated by the “localization of lipoproteins” (Lol) system. In *Escherichia coli*, the Lol system comprises five proteins where LolCDE —an ABC transporter— extracts lipoproteins from the IM and transfers them to the periplasmic chaperone LolA, which in turn shuttles them to the OM receptor LolB (Fig. 1). LolB, itself a lipoprotein, is anchored to the inner leaflet of the OM and inserts incoming lipoproteins into the bilayer^3,4^. As this pathway is essential for OM biogenesis, it represents an attractive target for the development of novel antibacterial agents. A compelling example is lolamicin, a small-molecule inhibitor of the LolCDE complex in certain Gram-negative bacteria, such as *E. coli*, which selectively spares commensal gut microbiota^5^.

Comparative studies reveal substantial variation in the Lol system across bacterial phyla. For example, *Helicobacter pylori* expresses a LolF homodimer in place of the LolCE heterodimer^6^, and while the insertase LolB is crucial for *E. coli* most bacteria appear to lack LolB entirely^7^. Some studies suggest that certain bacteria, such as *Caulobacter vibrioides* (α-proteobacteria), expresses a LolA protein with dual functions, being responsible for both transporting lipoproteins across the periplasm and inserting their acyl chains into the membrane^8^. Thus, although the overall process of lipoprotein transport is conserved, the molecular components differ, raising intriguing questions about the evolution and adaptability of this pathway.

*Porphyromonas gingivalis*, an oral pathogen implicated in periodontitis and systemic diseases such as Alzheimer’s and rheumatoid arthritis, belongs to the Bacteroidota phylum^9,10^. Its lipoprotein processing pathway diverges from that of enterobacteria. The canonical Lnt, responsible for adding the third acyl chain, has not been identified, yet many of its lipoproteins appear triacylated^11,12^, suggesting an alternative acylation enzyme. Indeed, recent studies have identified an enzyme in Bacteroidota, known as Lnb, which can add the third acyl chain—however its sequence and predicted structure is distinct from Lnt^13^. Furthermore, no LolB homolog was annotated in *P. gingivalis*, leaving the mechanism by which lipoproteins are integrated into the OM of this bacterium unresolved.

*P. gingivalis* possesses several proteins predicted to be lipoproteins. Among these are the components of its two distinct type-V fimbriae: Mfa1 (built up from Mfa1-5) and FimA (built up from FimA-E)^14^. With the exception of Mfa5^15^ these fimbrial proteins are synthesized as lipoprotein precursors, in which the conserved cysteine is acylated, after which they are transported to the OM^16^. After transport, Mfa2 and FimB, respectively, remain anchored to the OM via their acyl groups^17^, whereas in the other fimbrial proteins the N-terminus, including the acyl groups, is removed to allow incorporation into the fimbrial stalk or tip structure^14^. The mechanism by which these fimbrial proteins are transported across the periplasmic space and over the OM remains unknown.

With recent advances in protein structure prediction (e.g., AlphaFold)^18^, our possibility to detect distant homologs and explore their functions beyond simple sequence similarity has increased significantly. Motivated by this, we screened the *P. gingivalis* proteome for lipoproteins and were able to identify a long-searched candidate LolB-like protein (PGN0994). We characterized its crystal structure and its interactions with LolA (PGN0486), and small-molecule inhibitors, in comparison with previously studied LolB proteins^19,20^. In conclusion, our studies showed that the LolB-like protein indeed is the interaction partner of LolA, however its involvement in the transport of the lipidated fimbrial proteins remains unclear. Our findings expand the understanding of lipoprotein trafficking in Bacteroidota but also raises questions on the diversity of OM biogenesis pathways across Gram-negative bacteria.

## Materials and methods

### In silico search for LolB-like proteins

The protein sequences from *P. gingivalis* ATCC 33277 were searched for proteins containing a lipoprotein signal peptide using SignalP^21^. In short, a lipoprotein signal peptide contains a lipobox motif that ends with a cysteine that will be acylated. The sequences were subsequently used for structure prediction using Alphafold^18^ unless the structure was already known and deposited in the protein data bank. Next, structural relatives of the models were explored using DALI^22^.

### Bacterial strains, plasmids and growth conditions

Bacterial strains and plasmids used in this study are listed in Supplementary Table S1. *P. gingivalis* ATCC 33277 was cultivated anaerobically at 37 °C on Brucella HK agar (Kyokuto Pharmaceutical Industrial Co., Tokyo, Japan) supplemented with 5% (v/v) laked rabbit blood, 2.5 µg/mL hemin, 5 µg/mL menadione, and 0.1 µg/mL dithiothreitol (DTT). Anaerobic conditions were established using an ANOXOMAT system (Advanced Instruments, Norwood, MA, USA), with a gas mixture composed of 80% N_2_, 10% H_2_, and 10% CO_2_. Liquid cultures were grown in trypticase soy broth containing 0.25% (w/v) yeast extract, 2.5 µg/mL hemin, 5 µg/mL menadione, and 0.1 µg/mL DTT (sTSB). Where appropriate, the medium was supplemented with 20 µg/mL erythromycin. *E. coli* was cultured in Luria–Bertani (LB) medium supplemented, when necessary, with 100 µg/mL ampicillin or 50 µg/mL kanamycin.

### Cloning, expression and purification

The gene encoding a LolB-like protein, from now referred to as LolB-PG (GenBank BAG33513.1; PGN0994)^23^ was PCR amplified from genomic DNA of strain ATCC 33277. Primers were designed to exclude the signal peptide residues 1–27. The PCR product was digested with *NcoI/Acc65I* and ligated into equivalent sites of pET-His1a, in-frame with a histidine tag and a short linker (Supplementary Table S3) followed by residues 28–287. A shorter construct, truncated at the N-terminus, containing residues 41–287 was also made. Both versions of the proteins were expressed in *E. coli* BL21(DE3) in LB and 50 µg/mL kanamycin at 37 °C. At OD_600_ ~0.4, protein expression was induced with 0.5 mM Isopropyl β-d-1-thiogalactopyranoside (IPTG) and the temperature was lowered to 24 °C. After four hours the bacteria were harvested by centrifugation at 5300 x g and the pellets were stored at -80 °C until further use. The cell pellets were resuspended in lysis buffer (50 mM sodium phosphate buffer pH 7.6, 0.3 M NaCl and 10 mM imidazole supplemented with 1% triton-X100) and sonicated on ice. The lysate was centrifuged (63,000 x g for 20 min). The supernatant was passed over a column packed with His60 Ni-resin (Takara). The column was washed with lysis buffer containing 30 mM imidazole after which the protein was eluted with the same buffer containing 0.3 M imidazole. Next, the histidine tag was removed by incubation with ~ 1% (w/w) Tobacco Etch Virus (TEV) protease over night at + 4 °C. After buffer exchange (50 mM sodium phosphate pH 7.6, 0.2 M NaCl) the protein solution was passed over the affinity column again to remove histidine tags and the TEV protease. The flow through and wash fractions containing cleaved LolB-PG were collected and concentrated. The protein was further purified by gel filtration (HiLoadTM 16/60 Superdex™200 prep-grade column (GE Healthcare)) equilibrated with the same buffer. Fractions containing the peak of interest were concentrated in 20 mM HEPES pH 7.5, 0.2 M NaCl.

For the protein variant LolA3-PG, the encoding gene (BAG33466.1; PGN0947) was ordered from Thermo Fisher Scientific and codon optimized for expression in *E. coli*. The construct comprises residues 24–263 (excluding the signal peptide), in frame with a histidine tag and a short linker (Supplementary Table S3). The protein was expressed in BL21 (DE3) in LB supplemented with 100 µg/mL ampicillin at 37 °C. At OD_600_ ~0.6, protein expression was induced with 0.5 mM IPTG and the temperature was lowered to 24 °C. The protein was expressed for four hours before the bacteria were harvested. LolA3-PG was purified and the expression tag was removed using TEV protease following the same protocol as described for LolB-PG. LolA from *P. gingivalis* (LolA-PG) and from *Vibrio cholerae* (LolA-VC) were purified as described previously^19^.

### Crystallization and structure determination of LolB-PG and LolA3-PG

Crystallization screening was performed by the sitting-drop vapor-diffusion method at 20 °C in 96-well MRC-crystallization plates (Molecular Dimensions). Droplets of 0.2 µl LolB-PG protein (18 mg/ml) or LolA3-PG (15 mg/ml) were mixed with 0.1 µl of screening solutions from Molecular Dimensions using a Mosquito (TTP Labtech) pipetting robot. The crystals were flash cooled in liquid nitrogen and transported to the European Synchrotron Radiation Facility (ESRF), where diffraction data of LolB-PG and LolA3-PG were collected at beamlines ID30B and ID23-2 respectively. The diffraction data were automatically scaled with XDS^24^ and processed with Aimless^25^. The structures were solved using molecular replacement in Phaser^20^ part of the Phenix program package^26^, using AlphaFold models as search models. The structure of LolB-PG was refined to 2.1 Å resolution using phenix.refine^27^ combined with rebuilding in Coot^28^. LolA3-PG was similarly refined to 2.3 Å. Data processing and refinement statistics are presented in Supplementary Table S2. The figures were prepared with CCP4mg^29^.

### Characterization of interactions using ITC

Isothermal titration calorimetry (ITC) experiments were performed with a MicroCal auti-iTC200 instrument at 25 °C in 20 mM HEPES pH 7.5, 150 mM NaCl. Total injections were 20 of 2 µl, except the first, which was 0.4 µl, and occurred every 150 s. Reference power was 10 µcal/s and initial delay of 60 s. For LolB-PG, LolA-PG, LolA3-PG, LolA-VC and Polymyxin B/colistin/nonapeptide/A22 the interaction stirring speed was 1000 rpm. For protein and Polymyxin derivatives, control experiments were performed by injecting ligands with concentrations used in test, on buffer. Corresponding data were subtracted from respective interactions as a linear fit. Raw data were fitted with MicroCal PEAQ-ITC analysis software using single site binding model for all the data sets. Polymyxin B, colistin, nonapeptide and A22 were obtained from Sigma-Aldrich. All measurements were performed in triplicates.

### Characterization of interactions using BLI

Biolayer interferometry (BLI) experiments were performed with an OctetR2 Protein Analysis system (Sartorius) using Ni-NTA biosensors. The ligand, His-tagged LolB-PG, (1 µM), and the analyte, LolA-PG without His-tag, (from 1.5625 µM to 100 µM), were prepared in a buffer containing 20 mM HEPES and 150 mM NaCl. Experiments were conducted at 25 °C with 1000 rpm shaking in 96-well microplate. The binding kinetics assay adopted the following sequential steps – baseline: biosensors were equilibrated for 30 s, loading: the ligand, was immobilized for 600 s, a quenching step was performed for 120 s, regeneration/washing was performed with 1 M salt (High salt) for 30 s followed by a wash with buffer (Low salt) for 30 s. This was followed by repeated cycles for each analyte concentration: baseline equilibration for 60 s, association with analyte for 120 s, dissociation for 120 s, regeneration/washing. Data were processed using Octet Analysis Studio, measuring binding association (Ka), dissociation kinetics (Kd) and affinity constants (K_D_). All measurements were performed in triplicates.

### Modelling of a LolA-LolB complex

The complexes between LolB–LolA and LolB–LolA3 from *P. gingivalis* were modelled using AlphaFold3^30^. In short, sequences of the proteins, without their respective signal peptide, were used as input.

### Construction of LolB-PG deletion mutant (Δ*pgn0994*) in *P. gingivalis*

Construction of the *P. gingivalis* deletion mutant was performed using a PCR-based overlap extension strategy, following established protocols described previously^31^. DNA fragments that allowed the replacement of the *pgn0994* gene with the *ermF* gene, encoding an *ermF–ermAM* cassette, in the *P. gingivalis* chromosome were constructed. The primers and their overlapping regions of *ermF* are shown in Supplementary Table S3. Briefly, *ermF* was amplified from the ATG start codon to the TAA stop codon with primers ErmFFw and ErmFRv to generate a 1,084-bp product from pVA2198. For construction of the *pgn0994* deletion cassette, the flanking sequence upstream of *pgn0994* was amplified with er1F and er1R, which have homology to the 5’ end of the *ermF* fragment. The flanking sequence downstream of *pgn0994* was amplified with er2F and er2R, which have homology to the 3’ end of the *ermF* fragment. The *ermF* and *pgn0994*-upstream and *pgn0994*-downstream fragments were used as templates for overlap extension PCR to generate a deletion cassette in which *pgn0994* was replaced by *ermF*.

The deletion cassette created was ligated into pCR-Blunt II-TOPO according to the manufacturer’s directions and the resulting recombinant plasmids were cloned in *E. coli* TOP10. The plasmid was introduced into *P. gingivalis* competent cells by electroporation. After 24 h of anaerobic incubation in sTSB, the cells were plated on Brucella HK agar supplemented with 5% (v/v) laked rabbit blood, 2.5 µg/ml hemin, 5 µg/ml menadione, 0.1 µg/ml DTT and 20 µg/ml erythromycin and the plates were incubated anaerobically at 37 °C for 7 days. Recombination was confirmed by PCR analysis and DNA sequencing.

### Subcellular fractionation

*P. gingivalis* cells were washed with 10 mM HEPES (pH 7.4) containing 0.15 mM NaCl and then resuspended with 10 mM HEPES (pH 7.4) containing 0.1 mM N-α-p-tosyl-l-lysine chloromethyl ketone, 0.2 mM phenylmethylsulfonyl fluoride and 0.1 mM leupeptin. The cells were disrupted by sonication, and the remaining undisrupted bacterial cells were removed by centrifugation at 1000 x g for 10 min. The supernatant was used as the whole cell lysate (WCL). The envelope (Env) fraction was collected as a pellet by centrifugation of the whole cell lysate at 100,000 x g for 60 min at 4 °C. The supernatant was used as the soluble (Sol) fraction. The OM and IM fractions were separated by the differential detergent extraction method from envelope fraction with 1% Triton X-100 in HEPES buffer containing 20 mM MgCl_2_ for 30 min at 20 °C. The culture supernatant (Sup) was prepared by collecting the growth medium from bacterial cultures, followed by centrifugation at 1000 × g for 10 min to remove cells and debris. The supernatant was then filtered through a 0.22 μm filter. Proteins in the supernatant were concentrated by ammonium sulfate precipitation: solid ammonium sulfate was added to 70% saturation, and the mixture was incubated on ice for 1 h. Precipitated proteins were collected by centrifugation at 12,000 × g for 30 min at 4 °C, resuspended in HEPES buffer.

### SDS-PAGE and immunoblotting

The cell-fractionated protein samples containing 5 µg of total protein were solubilized in SDS sample buffer supplemented with 2-mercaptoethanol and heated at either 100 °C for 5 min or 75 °C for 5 min. The proteins were then separated by SDS–PAGE and electroblotted onto PVDF membranes. Membranes were blocked with 5% skim milk and subsequently incubated with primary rabbit polyclonal antibodies against purified Mfa1, FimA fimbriae, Mfa2, or Mfa3^32^. After washing, membranes were incubated with HRP-conjugated goat anti-rabbit IgG (MP Biomedicals, Santa Ana, CA). Immunoreactive bands were visualized using Western Blot Chemiluminescence HRP Substrate (Takara Bio Inc., Otsu, Japan).

## Results

### Structure determination and comparison

**Fig. 2:**
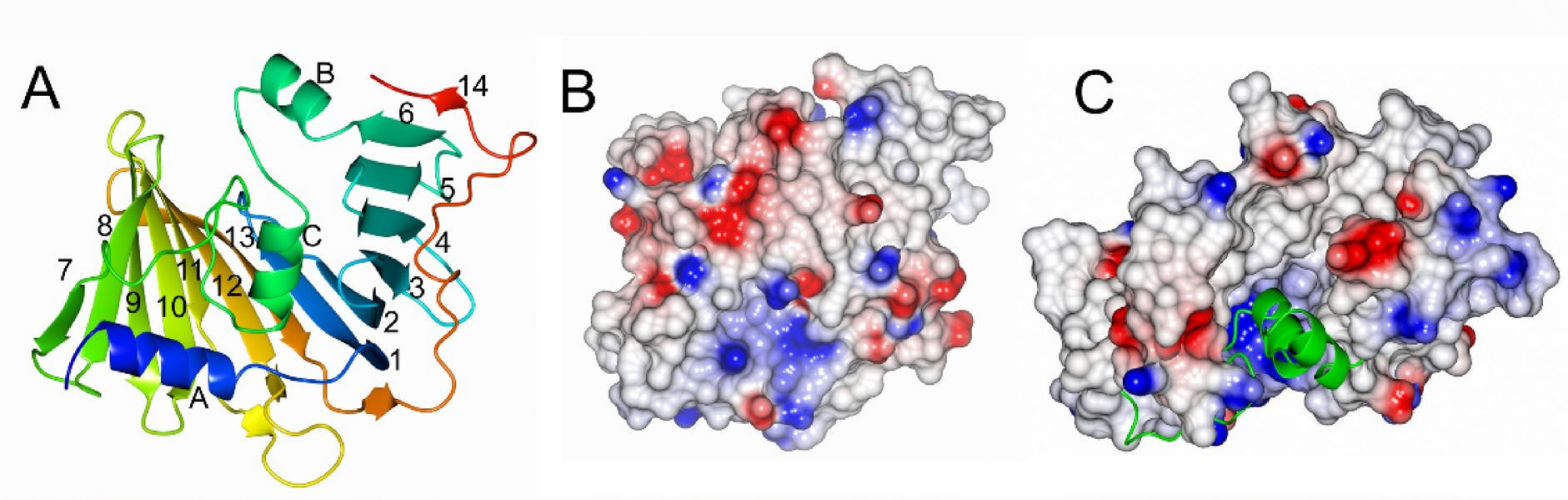
The overall structure of LolB-PG. (**A**) LolB-PG is shown as a ribbon model, with the color blending from blue (N-terminus) to red (C-terminus). The curved β-sheet consists of 14 strands forming a binding cleft, which is protected by helices B and C. (**B**) Electrostatic surface representation of LolB-PG in the same orientation as A. (**C**) Electrostatic surface representation of LolB-PG with a view down the cleft where helices B and C are depicted as ribbon models in green. Positive potential is shown in blue and negative potential in red. Notably, the region around the cleft, or mouth, is predominantly uncharged.

The structure of the protein encoded by *pgn0994*, was modelled by Alphafold and identified as structurally related to LolB from *E.coli* (PDB 3wjv)^33^ with a Z-score of 12.8. LolB-PG was cloned, overexpressed and purified. A truncated version of the protein, starting at residue 41 and lacking the flexible N-terminal region, exhibited improved stability and higher yields during expression and purification. Consequently, this construct was selected for all downstream analyses and applications. Protein crystals were obtained in the MEMGOLD2 screen in condition 30 (50 mM Zinc acetate, 50 mM MES pH 6.5, 11% PEG 8000). Diffraction data were collected and the structure was solved using molecular replacement and refined to 2.1 Å resolution. The structure consists of a predominantly antiparallel curved β-sheet with 14 strands. The sheet forms a hydrophobic cleft, with its base composed of helix A. The groove is shielded by helices B and C and an extended region following helix C (Fig. 2A). Electrostatic maps reveal that the protein is mainly non-polar with no significant areas of positive or negative charges, and the sides of the cleft are mainly hydrophobic whereas the bottom is positively charged (Fig. 2B, C).

Dali^22^ was used to search for related structures. As with the original AlphaFold model the closest hit was a protein from *Vibrio parahaemolyticus*, classified as a potential lipoprotein sorting protein (PDB ID: 3bk5, Z-score: 16.3, 14% sequence identity). This was followed by an uncharacterized protein from *Desulfovibrio piger* (PDB ID: 4z48, Z-score: 16.3, 8% sequence identity) and LolB from *E. coli* (PDB ID: 1iwm, Z-score: 15.4, 14% sequence identity)^20^.

**Fig. 3:**
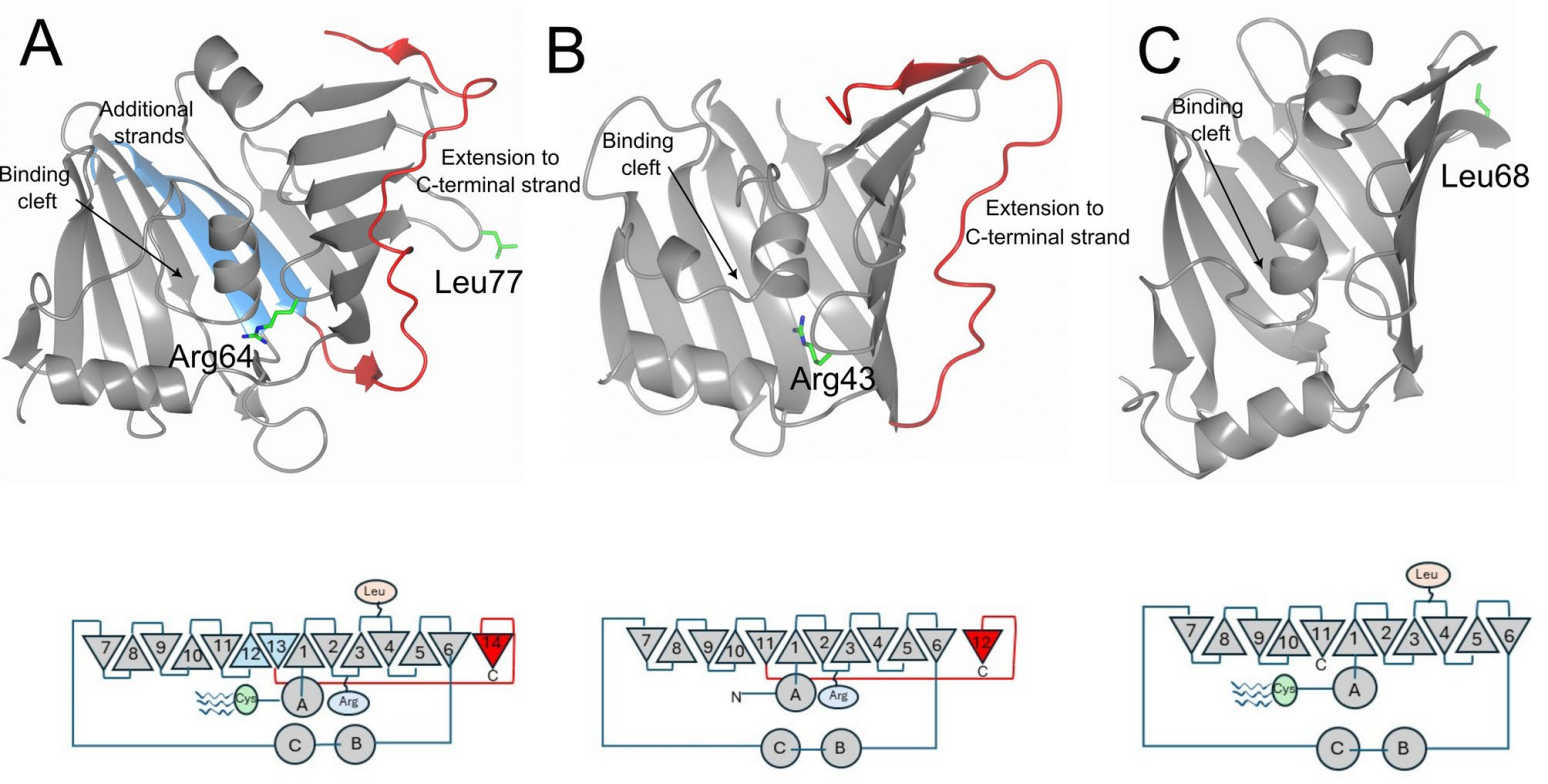
LolB-PG compared to LolA-EC and LolB-EC. (**A**) LolB-PG is significantly larger than LolA-EC (**B**) and LolB-EC (**C**), which is a result of two additional strands in the β-sheet, in this figure depicted in blue. LolB-PG also exhibits several characteristics shared with either LolA-EC or LolB-EC. A, (**B**) The LolA-like features include the extended segment along the convex side of the β-sheet, ending in the final β-strand (illustrated in red), and the presence of an inward-pointing arginine on the loop between β2 and β3. A, (**C**) The LolB-like features include an N-terminal cysteine that can be acylated and anchored to the membrane and an exposed leucine on the loop separating β3 and β4. The side chains are depicted as sticks. Each protein is also depicted as a topology diagram where strands are shown as triangles and helices as circles. The position of the conserved arginine, leucine and acylated cysteine are indicated.

LolB-PG exhibits several characteristic features resembling both LolB and LolA from *E. coli*^20^(LolB-EC and LolA-EC respectively). The LolB characteristics include the presence of a cysteine as the first amino acid after the signal peptide, indicating that the mature protein will be acylated and anchored to a membrane. LolB-EC has a conserved Leu (Leu68) exposed on the loop connecting β3 and β4. This Leu has been proven important for the delivery of lipoproteins to the OM^33^. LolB-PG has similarly an exposed Leu (Leu77) on the equivalent loop (Fig. 3).

Interestingly, LolB-PG also has several LolA-like features, including an extended segment running on the convex side of the sheet, originating from the penultimate strand in the center of the sheet and extending to the edge where it forms the terminal β-strand, that runs in parallel with β6. LolA-EC (but not LolA-PG) also has a well-studied Arg-Pro motif on the loop between β2 and β3 (Arg43). The side chain of the arginine points inward contributing to the closure of the groove. This arginine has also been shown to be important for lipoprotein binding and the transfer from LolA to LolB. Intriguingly, LolB-PG has an arginine (Arg64) in the same position as the Arg-Pro motif in LolA-EC and the arginine forms hydrogen bonds to β1 and helix C, thereby closing the cleft in a similar manner.

A significant difference when comparing LolB-PG with both LolA-EC and LolB-EC is the size of the protein: 29 kDa, which is substantially larger than LolA-EC (20 kDa) and LolB-EC (21 kDa). This is a consequence of that LolB-PG has two additional strands in the β-sheet compared to LolA-EC and LolB-EC (Fig. 3). This also immediately affects the size of the binding cleft, with LolB-PG having a binding cleft of 1790 Å^3^ compared to LolB-EC (27 Å^3^) and LolB-VC (11 Å^3^) as calculated with CastP^34^. The accessibility of the binding cleft is a result of that both LolA-EC and LolB-EC have a helix (helix B) that is tilted into the binding cleft, whereas the corresponding helix in LolB-PG is positioned in a way that leaves the binding cleft fully open. Flexibility within the helix B–helix C region can likely contribute to variability in the size of the binding clefts.

### Comparison between LolA and LolA3

While this manuscript was in preparation, additional LolA and LolB proteins were reported and characterized in the bacteroidota species *Flavobacterium johnsoniae*^35^. Their study also identified a LolA protein in *P. gingivalis*, (LolA3), in addition to the LolA and LolB proteins we were examining (in their study referred to as LolA1 and LolB1)^35^. To determine whether LolB-PG interacts with the LolA proteins, we cloned the gene encoding LolA3, and purified the protein in order to study its structure and function.

LolA3-PG crystals were obtained in several conditions in the MORPHEUS screen and a crystal from condition G5 (0.2 M Sodium formate, 0.2 M Ammonium acetate, 0.2 M Sodium citrate tribasic dihydrate, 0.2 M Potassium sodium tartrate tetrahydrate, 0.2 M Sodium oxamate, Sodium HEPES; MOPS, pH 7.5, 40% v/v PEG 500 MME, 20% w/v PEG 20000) was used for data collection. The LolA3-PG structure consists of an antiparallel β-sheet with 13 strands. The sheet forms a hydrophobic groove, and as in LolB-PG, helix A forms the base of the cleft and the additional helices B and C shield the groove. There is also a fourth short helix in the C-terminus that packs against the convex side of the β-sheet (Supplementary Figure S1).

LolA-PG and LolA3-PG share similar overall structures and calculated isoelectric points (9.3 and 9.2 respectively; LolB-PG 8.6) but exhibit low sequence identity (22%). LolA3-PG is larger than LolA-PG (28 kDa vs. 21 kDa) and the most significant structural difference is that the C-terminus of LolA3-PG does not form the last β-strand of the sheet but instead short α-helix (helix D). Additionally, the β-sheet is larger and contains two additional strands (β12 and β13) which make the topology of the sheet more similar to LolB-PG than to LolA-PG. Electrostatic surface analysis indicates that LolA-PG has a higher density of negatively charged residues^19^, whereas LolA3-PG displays more neutral and positive residues on its surface (Supplementary Figure S1). LolA3 has neither a leucine on its β3β4 loop (a LolB feature) nor an arginine on the β2β3 loop (a LolA feature).

### Interactions between LolA, LolA3 and the LolB protein

Lipoproteins are proposed to be transferred from LolA to LolB through a “mouth to mouth” mechanism in which the acyl chains of the lipoprotein slide from the binding cleft of LolA to the cleft of LolB^36^. Previous work has shown that LolA and LolB interact also in the absence of lipoprotein. In *E. coli* the dissociation constant (Kd) for the LolA-LolB pair has been reported as 30.6 µM^37^ and in *V. cholera* it was measured to 30.2 µM^19^. To assess whether our identified LolB-like protein from *P. gingivalis*, LolB-PG, interacts with LolA-PG, or the paralog LolA3-PG, we performed ITC measurements. We show that LolB-PG bound to LolA-PG with a Kd of 10 µM and similar results were obtained with BLI (6.2 µM). In contrast, only very weak interaction was detected between LolB-PG and LolA3-PG. This observation is consistent with the complementary surface charge distributions of LolA-PG (predominantly negative) and LolB-PG (partly positive), in contrast, LolA3-PG exhibits mainly positive surface potentials around its barrel opening (Fig. 2, Supplementary Figures S1 and S2).

**Fig. 4:**
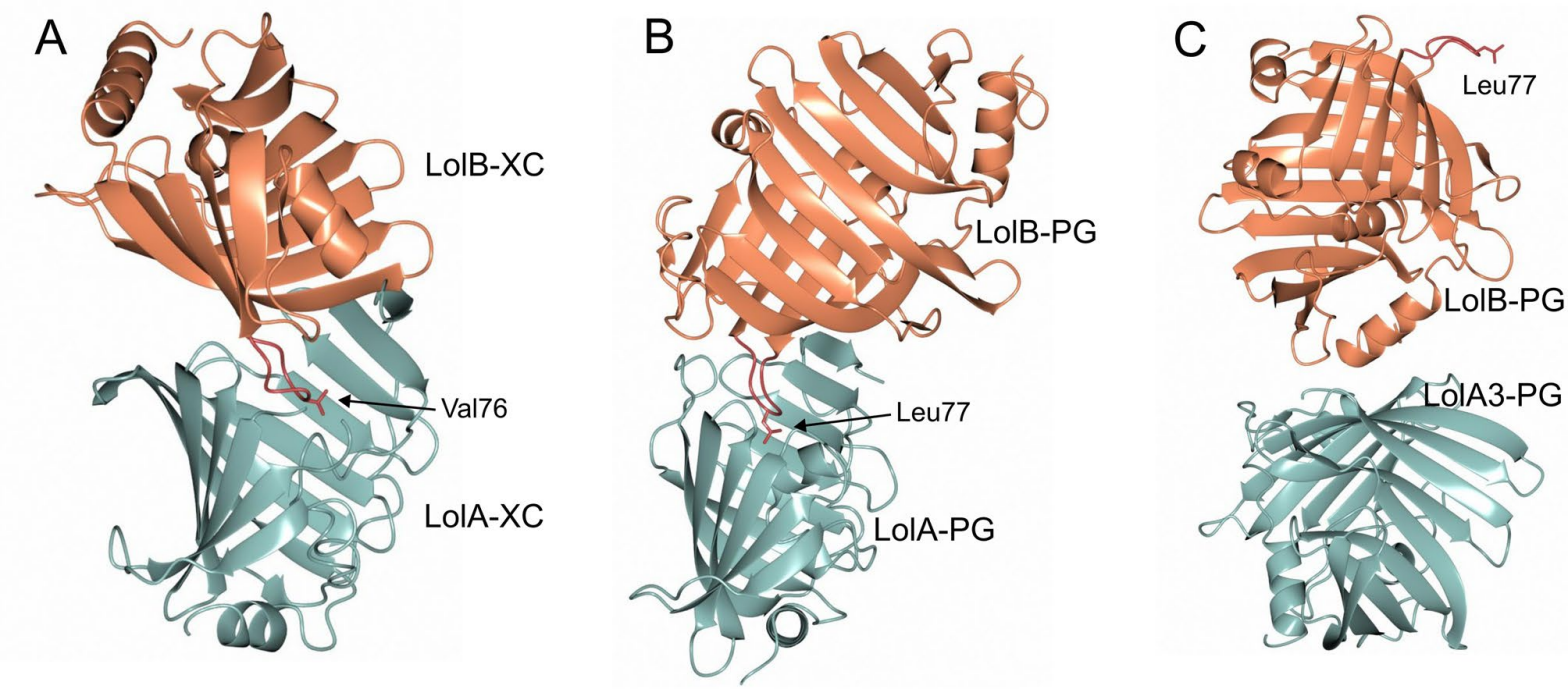
Complexes between LolB and LolA. LolA and LolB pairs can interact in the absence of a bound lipoprotein. (**A**) The crystal structure of the complex between LolA and LolB from *X. campestris*^38^(LolA-XC and LolB-XC). (**B**) The complex between LolB-PG and LolA-PG was modelled using AlphaFold3. In both complexes the two binding clefts face each other mouth-to-mouth with the β3β4 loop of LolB inserted into the binding cleft of LolA. (**C**) The modelled complex between LolB-PG and LolA3-PG indicates no direct mouth-to-mouth interaction. The hydrophobic residue located on the β3β4 loop is indicated (Leu77 in LolB-PG and Val76 in LolB-XC) LolA proteins are shown in sea green and LolB in coral.

Similarly, LolB-PG failed to interact with LolA homologs from another bacterial species (*V. cholerae*; Table 1, Supplementary Figure S4G) further supporting the specificity of the LolA-LolB interaction. This conclusion is reinforced by structural modelling with AlphaFold3^18^, which produces a mouth-to-mouth interaction between LolB-PG and LolA-PG where the LolB-PG β3β4 loop, including Leu77, is inserted into the LolA-PG binding cleft (Fig. 4B). This complex is very similar to the experimentally determined LolA-LolB complex of *Xanthomonas campestris*^38^ of which Val76 on the β3β4 loop is inserted and the Kd was measured to 14.6 µM (Fig. 4A). In contrast, modelling of the interaction between LolB-PG and LolA3-PG results in a complex in which the two cleft-openings do not interact (Fig. 4C).

**Table 1:** Protein-protein interactions.

Protein pairs	Kd (ITC)	Kd (BLI)
LolB-PG/LolA-PG	10 µM	6.1 µM
LolB-PG/LolA3-PG	Weak interaction	
LolB-PG/LolA-VC	No binding	

### Interactions between the LolB-PG protein, polymyxins and A22

**Fig. 5:**
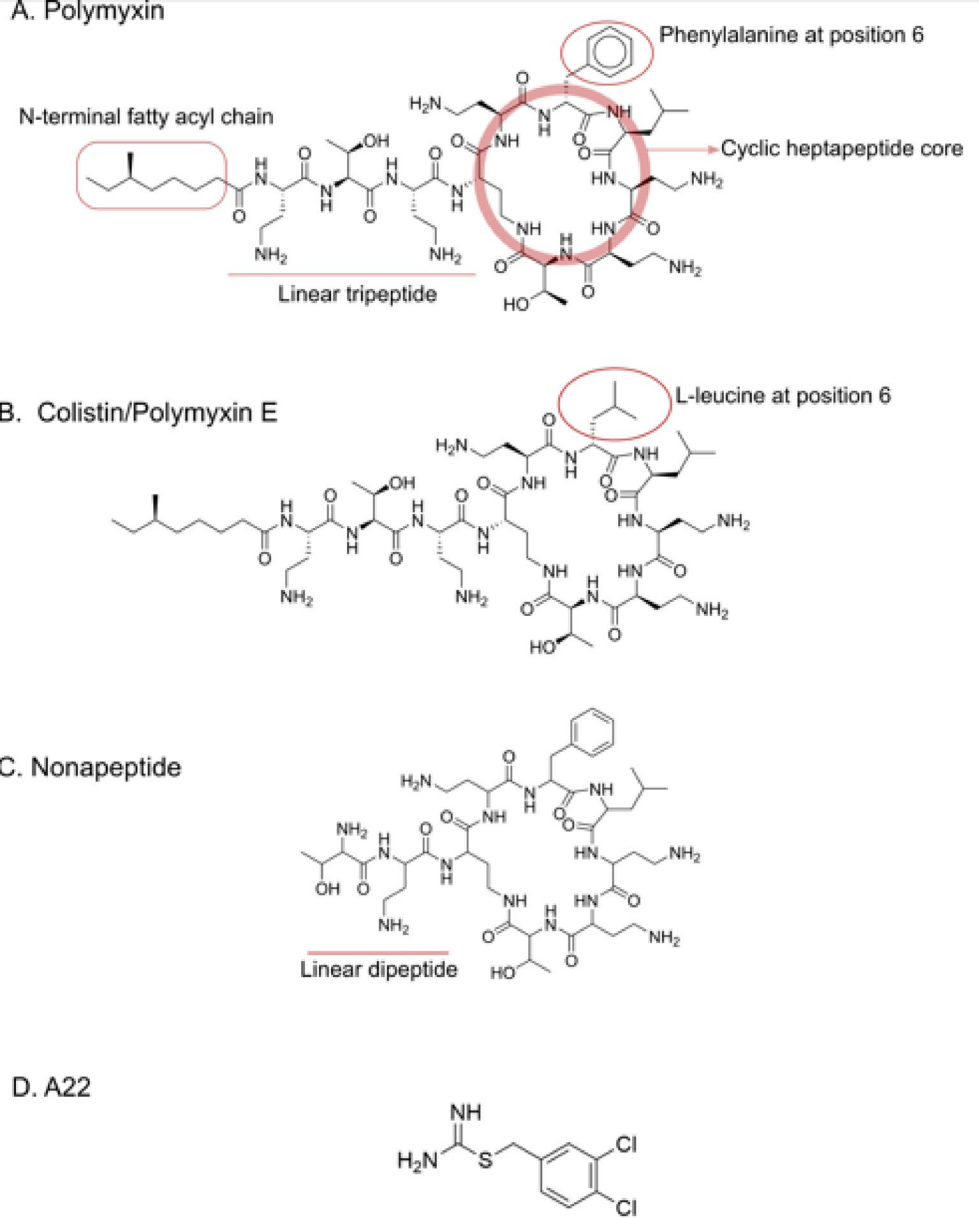
Structures of Polymyxins and A22. Chemical structures of (**A**) Polymyxin, (**B**) Colistin (Polymyxin E) (**C**) Nonapeptide and (**D**) A22. Polymyxin and Colistin are very similar molecules consisting of a cyclic heptapeptide core, a linear tripeptide and an N-terminal acyl chain. The difference between them is that a phenylalanine in the polymyxin heptapeptide is replaced by a leucine in the Colistin molecule. Nonapeptide has the same heptapeptide core as polymyxin but has a shorter peptide tail and lacks the acyl chain. A22 is a degradation product of an antibacterial substance.

Polymyxins (Polymyxin B and colistin (also called Polymyxin E)) are last resort antibiotics to treat Gram negative infections. They consist of a cationic cyclic heptapeptide and a lipid tail (Fig. 5). We have previously shown that LolA from *P. gingivalis* and *V. cholerae*, but not *H. pylori*, bind polymyxins whereas LolB from *V. cholerae* does not^19,39^. Similarly the LolB-PG protein did not exhibit any interactions with polymyxins at the concentrations used in the experiments. However, LolB-PG demonstrated weak binding to A22, a degradation product of the antibacterial substance MAC13243^40^, with a Kd of 0.4 mM. This weak interaction is comparable to the interactions observed between A22 and LolA-PG, LolA-VC, and LolB-VC^19^ (Table 2; Fig. 5, Supplementary Figure S4). In summary, LolB from *P. gingivalis* does not bind polymyxins and exhibits only weak, non-specific interaction with A22, consistent with previous observations for Lol proteins; this suggests limited relevance as an antibiotic target for these compounds.

**Table 2:** ITC data for *P. gingivalis* LolB and LolA3 with A22, Colistin, Polymyxin B and nonapeptide.

Protein	Kd (A22)	Kd (Colistin)	Kd (Polymyxin B)	Kd (Nonapeptide)
LolB-PG	400 µM	No binding	No binding	No binding
LolA3-PG	66 µM	No binding	No binding	No binding

### Impact of LolB-PG for polymerization of type-V fimbrial proteins in *P. gingivalis*

To assess whether LolB-PG is required for the transport of type-V fimbrial proteins—which are synthesized as lipoprotein prior to their incorporation into the growing fimbriae—we generated a *P. gingivalis* mutant in which the *pgn0994* gene was replaced by *ermF* through allelic exchange generating a LolB deficient strain. The successful isolation of the Δ*pgn0994* strain indicated that this gene is not essential under the conditions tested. To assess whether the deletion of *pgn0994* affects the assembly, localization, or maturation of Mfa1- and FimA fimbrial components, we analyzed whole-cell lysates and subcellular fractions from wild-type *P. gingivalis* 33277 and Δ*pgn0994* by immunoblotting. First, the polymerization state of Mfa1 was examined under two denaturing conditions. Heating at 100 °C resulted in complete dissociation of the fimbrial structure into monomers, whereas heating at 75 °C caused only partial dissociation, allowing detection of polymeric forms. Immunoblot analysis under these conditions showed no noticeable differences in the presence of Mfa1 monomers or polymers between the wild type and the mutant (Supplementary Figure S5A). A similar analysis of FimA also demonstrated comparable polymerization patterns between the two strains (Supplementary Figure S5B). We next investigated the subcellular localization of the anchor protein Mfa2 using cell-fractionated protein samples. Only trace amounts, close to the detection limit, were detected in the soluble fraction (Supplementary Figure S5C). In contrast, clear signals were observed in the envelope fraction. This shows that Mfa2 indeed has been transported over the periplasm and is linked to OM, presumably by its acyl groups. This is consistent with our previous report of Mfa2 localization^17^. No difference in localization was observed between the wild-type and mutant strains, hence LolB-PG is not crucial for Mfa2 transport. Finally, the localization and maturation status of Mfa3 were examined. Consistent with our previous findings, an immature form of Mfa3—visible as a slightly higher–molecular-weight band—was detected specifically in the IM—likely representing the lipidated precursor prior to maturation (Supplementary Figure S5D). Both the wild-type strain and Δ*pgn0994* exhibited the same distribution and maturation patterns of Mfa3. These results indicate that LolB-PG is not involved in the transport of fimbrial proteins, at least not FimA and Mfa1–3 in *P. gingivalis* under these growth conditions.

## Discussion

To develop new antibiotics, it is essential to map both the conserved features and the diversity of bacterial pathways. The Lol system is a striking example: while the core function of lipoprotein transport is conserved, the molecular components vary considerably. Differences include whether lipoproteins are di- or triacylated, if the transport involves a LolCE heterodimer or a LolF homodimer, and whether or not lipoproteins are received at the outer membrane by LolB^6,41^. The next step of the transport also remains an open question: how some of the lipoproteins are translocated across the OM and exposed on the surface. Comparative genomic analyses have shown that whereas β- and γ-proteobacteria invariably encode a single *lolB* adjacent to *ispE* and *hemA*, this gene organization is absent in other phyla^8^, therefore Bacteroidota have been thought to lack LolB entirely^7^. Nevertheless, alternative proteins may have evolved to meet LolB-like functions^8^. In support, De Smet et al. (2025) recently showed that *F. johnsoniae*, another Bacteroidota species, encodes multiple LolA and LolB homologs with specialized functions in gliding motility and Type IX secretion^35^. Such modular diversification suggests that the Lol machinery has evolved lineage-specific adaptations that cover differences in cell envelope architecture and secretion systems.

*P. gingivalis* has a simplified version of this system: one functional LolB (LolB-PG), one primary LolA (LolA-PG), and a secondary paralog (LolA3-PG) of distinct structural and electrostatic character^35^. For comparison it should be noted that *P. gingivalis* has a functional Type IX secretion system but is considered to be non-gliding^42^ which may be the explanation for less need of multiple LolA and LolB variants.

Our crystal structure of LolB-PG revealed a protein that retains the characteristic β-sheet curvature and hydrophobic binding cleft typical of LolB from *E. coli*, yet exhibits notable differences in size, topology, and surface charge. LolB-PG possesses a curved 14-stranded β-sheet and an enlarged binding cleft (~ 1,790 Å³) compared with the much smaller cavities in *E. coli* and *V. cholerae* LolB^19^. This larger and more open binding cleft may suggest altered substrate or partner recognition. The absence of strongly charged patches around the binding cleft is consistent with a broader or more hydrophobic binding repertoire, potentially reflecting adaptation to distinct lipoproteins or periplasmic conditions in *P. gingivalis.*

Biophysical data further support a specific interaction between LolB-PG and LolA-PG, with dissociation constants in the low-micromolar range (10 µM by ITC; 6 µM by BLI), comparable to LolA–LolB pairs characterized in *E. coli*, *X. campestris*, and *V. cholerae*^19,37,38^.

It may be questioned whether it is relevant to study the interaction between LolA and LolB in isolation as the physiological interaction also involves the lipoprotein that is transferred from LolA to LolB. Most lipoproteins appear to contain a flexible linker between the N-terminal acylation site and the globular domain of the protein. This suggests that only a limited portion of the lipoprotein may contribute directly to the interaction between LolA and LolB during transfer. At the same time, the interaction between LolA and LolB must be sufficiently robust to mediate the transfer of a wide range of lipoproteins transported by the Lol system.

AlphaFold3 modelling of the LolA-PG/LolB-PG complex produced a “mouth-to-mouth” orientation similar to the experimentally determined complex for *X. campestris*, reinforcing the conservation of this interaction mode. In contrast, LolB-PG showed only weak or negligible binding to LolA3-PG, supporting the notion that LolA3 has a separate function in the bacterial cell. It may be of interest to investigate whether LolA3 exhbits dual LolA and LolB functions similar to LolA from *C. vibrioides*^8^(LolA-CV). The two proteins only share 24% sequence identity, and the structural features of LolA3 suggest possible functional divergence, as it lacks both the conserved arginine in the β2β3 loop and an exposed leucine residue typically associated with LolB function.

Collectively, these findings establish LolB-PG as the cognate partner of LolA-PG and suggest that *P. gingivalis* preserves a specific LolAB transport pair.

The discovery of LolB-PG also invites reconsideration of the broader evolutionary trajectory of the Lol pathway. Despite extremely low sequence identity (8–14%) among LolB homologs, their overall structures remain remarkably conserved, emphasizing that fold preservation, rather than sequence conservation, dictates function. This conservation amidst sequence divergence may underlie variable antibiotic sensitivities across species, as subtle changes in surface electrostatics can alter interactions with amphipathic compounds such as polymyxins.

Phenotypic analysis of the Δ*pgn0994* mutant revealed no measurable differences in the polymerization, maturation, or localization of major fimbrial components (Mfa1, Mfa2, Mfa3, and FimA). Immunoblot analyses under denaturing and mild conditions confirmed equivalent polymeric and monomeric forms in wild-type and the mutant strain, indicating that LolB-PG is not required for the assembly or membrane anchoring of these fimbrial proteins. This observation mirrors findings in *F. johnsoniae*, where deletion of *lolA1* or *lolB1* had minimal morphological consequences under nutrient-rich conditions, although loss of *lolA1*—but not *lolb1*—affected gliding and cell shape when grown in minimal media^35^. A similar observation was observed in *E. coli* where it was shown that both LolA-EC and LolB-EC could be bypassed under certain conditions^43^. The lack of phenotype change in *P. gingivalis* therefore appears consistent with the possibility that LolB-PG operates redundantly or that fimbrial precursors utilize a Lol-independent export route.

The absence of a discernible phenotype should be interpreted in the context of the complex, multistep maturation of fimbrial proteins. After periplasmic transport, these lipoproteins must cross the outer membrane and become exposed to membrane bound gingipains that remove the acylated N-terminal extension before polymerization into mature fimbriae^44^. This sequence of processing events implies that additional, specialized machineries may substitute for Lol-mediated delivery. It is plausible that elements of the Type IX secretion system or other periplasmic chaperones facilitate the outer-membrane translocation of these surface-exposed lipoproteins. In fact, many lipoproteins in Bacteroidota are surface-exposed^45,46^, which implies several routes for lipoprotein transfer. Hence, the lack of effect from *pgn0994* deletion does not exclude an essential role for LolB-PG in other trafficking contexts.

The electrostatic properties of LolA and LolB proteins appear to influence their interaction with lipidated antibiotics and perhaps with native substrates. Our findings show that LolA-PG, but not LolB-PG, binds weakly to polymyxins, and that LolB-PG interacts only minimally with A22, similar to observations in LolA-VC, LolB-VC and LolA-HP^19,39^. The differential binding correlates with the surface charge of the proteins: negatively charged surfaces, as in LolA-PG, favour binding of cationic peptides such as polymyxins, whereas the largely neutral or hydrophobic LolB-PG surface does not. These differences emphasize that homologous proteins within the Lol pathway can differ substantially in their chemical interactions, an important consideration for drug development targeting lipoprotein trafficking.

Further research is needed to identify the lipoproteins that are trafficked by LolB-PG and to identify the sequence or structural determinants guiding their membrane targeting. Comparative proteomic analyses of wild-type and *Δpgn0994* strains under different growth conditions could reveal specific substrates dependent on this pathway. Moreover, examination of the short sorting motif immediately downstream of the acylated cysteine may help classify lipoproteins according to their final destinations—IM, inner leaflet of the OM, or outer leaflet. Such information would refine our understanding of lipoprotein function and clarify how *P. gingivalis* distinguishes between surface-exposed and periplasmic lipoproteins. Our findings collectively broaden the conceptual framework of lipoprotein trafficking and provide a basis for exploring the structural diversity of Lol proteins as potential antibacterial targets.

## Supplementary Information

Below is the link to the electronic supplementary material.

Supplementary Material 1

## Data Availability

Data supporting the finding of this manuscript are available from the corresponding author upon request. The atomic coordinates and structure factors for LolB-PG and LolA3-PG (9TPM and 9TP6) have been deposited in the Protein Data Bank (www.rcsb.org).
