# Supplementary material for: Identification of a LolB-like protein in Porphyromonas gingivalis reveals selective LolA–LolB pairing
Source: Sci Rep. 2026 Apr 22;16:13157. doi: 10.1038/s41598-026-49975-1 (PMC13103446; doi:10.1038/s41598-026-49975-1)
Supplement: Supplementary file 1 — Supplementary Material 1 [file 41598_2026_49975_MOESM1_ESM.pdf]

Supplementary information

Identification of a LolB-like protein in *Porphyromonas gingivalis* reveals selective LolA–LolB pairing

Deepika Jaiman[1,2], Makoto Hirohata[3], Yoshiaki Hasegawa[3] and Karina Persson[1,2*]

[1]Centre for Microbial Research (UCMR), Umeå University, Umeå, Sweden

[2]Department of Chemistry, Umeå University, Umeå, Sweden

[3]Department of Microbiology, School of Dentistry, Aichi Gakuin University, Nagoya, Japan

* Corresponding author:

Karina Persson

E-mail: karina.persson@umu.se

Tel: +46-90-7865926

Running title: Structure of *Porphyromonas gingivalis* LolB

This PDF includes

Supplementary Tables S1 to S3

Supplementary Figures S1 to S9

Supplementary Table S1. Bacterial strains and plasmids.

Strain	Genotype or relevant characteristics	Source or reference
Porphyromonas gingivalis		
ATCC 33277	Wild-type, type strain	ATCC
Δ*pgn0994*	*pgn0994* (encoding LolB-like protein)-deletion mutant from ATCC 33277, Em^r	This study
Escherichia coli		
TOP10	Chemically competent cells	Invitrogen
BL21 (DE3)	Chemically competent cells	Protein Expertise Platform, Umeå University
Plasmids		
pCR-Blunt II-TOPO	Cloning vector, Ap^r	Invitrogen
pVA2198	Plasmid used for a drug cassette, *ermF-ermAM*, Em^r	Nishiyama et al. (2021)
pET-His1a	Expression vector, Km^r	Protein Expertise Platform, Umeå University
pET151	Expression vector, Cb^r	Thermo Fischer Scientific
pET-His1a-LolB-long	Residues 28–287 and N-terminal His-tag sequence (MKHHHHHHPMSDYDIPTTENLYFQGAM)	This study
pET-His1a-LolB-short	Residues 41–287 and His-tag sequence (MKHHHHHHPMSDYDIPTTENLYFQGAM)	This study
pET151-LolA3	Residues 41–287 and His-tag sequence (MHHHHHHGKPIPNPLLGLDSTENLYFQGIDPFT)	This study

Supplementary Table S2. Processing and refinement statistics.

Data processing			Refinement		
LolB-PG		LolA3-PG	LolB-PG		LolA3-PG
Wavelength (Å)	0.87313	0.87313	Resolution (Å)	47.3-2.10 (2.23-2.10)	41.03-2.32 (2.41-2.32)
Space group	$P6_522$	P32	No. reflections (work/test)	15930 (839)	29206 (1460)
Cell dimensions			R_{work} / R_{free} (%)	22.7/27.8	24.4/28.9
a, *b*, *c* (Å)	94.7 94.7.5 104.9	123.9 123.9 44.4	No. atoms		
α, β, γ, (°)	90.0, 90, 120	90.0, 90, 120	Protein	1873	5908
Resolution (Å) *	47.34-2.10 (2.17-2.10)	41.03-2.31 (2.35-2.31)	Ligand/ion	92	30
R_{merge}	0.248 (3.818)	0.095 (2.005)	Water	78	7
Rpim	0.080 (1.232)	0.045 (0.763)	*B*-factors (Å^2)		
$I / \sigma I$	9.4 (0.8)	10 (0.2)	Protein/ligands/water	54.0/67.8/51.9	91.8/97.3/68.1
Completeness (%)	99.9 (99.5)	100 (100)	R.m.s. deviations		
Redundancy	19.3 (19.3)	5.3 (5.4)	Bond lengths (Å)	0.003	0.002
CC1/2	0.998 (0.468)	0.996 (0.360)	Bond angles (°)	0.470	0.470
Molecules in a.u.	1	3	PDB code	9TPM	9TP6

Supplementary Table S3. Primers used in this study.

Primer	Sequence (5′–3′)*
er1F	GCGACCTTTGTTCTGTGCAA
er1R	AAGCAATAGCGGAAGCTATCTCCATCTATCTATGAAGTTT
er2F	AAAAATTTCATCCTTCGTAGTTCTTCTTTTCTGGGATATA
er2R	TTCGCCGTCTATGCAATCGA
ErmFFw	GATAGCTTCCGCTATTGCTTTTTTGCTCAT
ErmFRv	CTACGAAGGATGAAATTTTTCAGGGACAAC

*Underlining shows overlapping regions of 5′ or 3′ end of *ermF*.

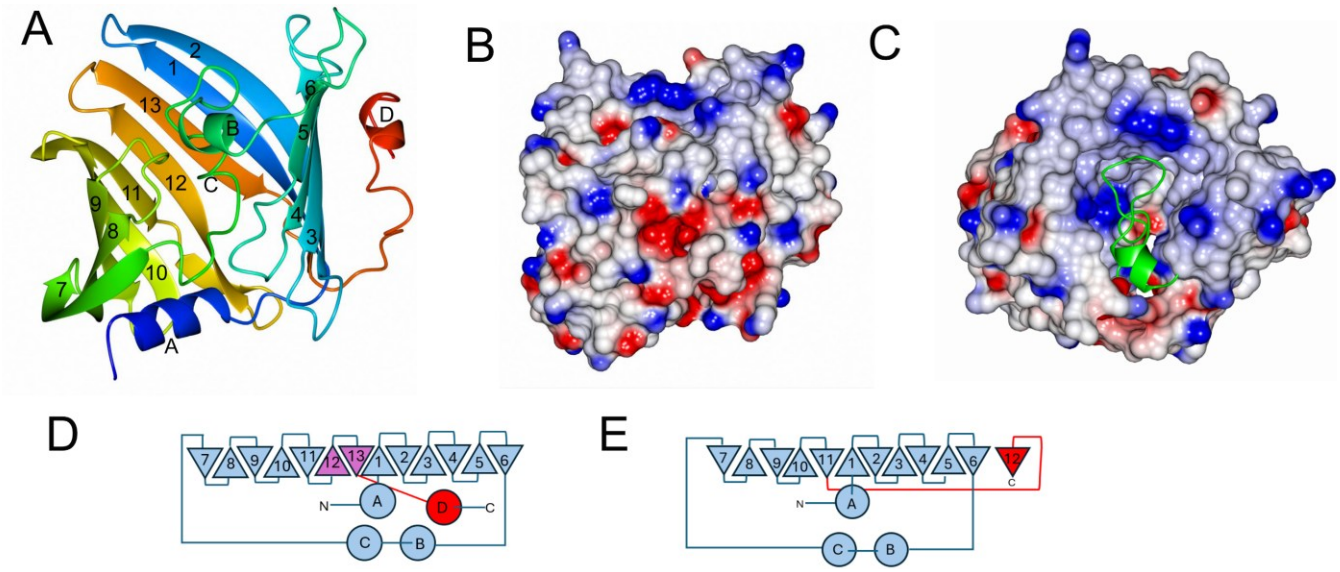

Supplementary figure S1.

Structure of LolA3-PG. A) A ribbon model of LolA3-PG colored from blue (N-terminus) to red (C-terminus). B) Electrostatic surface presentation in the same orientation as in A. C) Electrostatic surface presentation looking down the binding cleft. The segments that fill the cleft (Helix B and C) are depicted in ribbon style in green. The surface shows that the protein is predominantly positively charged around the mouth of the cleft. D) Topology diagram of LolA3-PG and E) LolA-PG where β-strands are depicted as triangles and helices as circles. The C-terminal helix of LolA3-PG and the C-terminal β-strand of LolA-PG are shown in red. The two strands in LolA3-PG that have no counterpart in LolA-PG are depicted in purple.

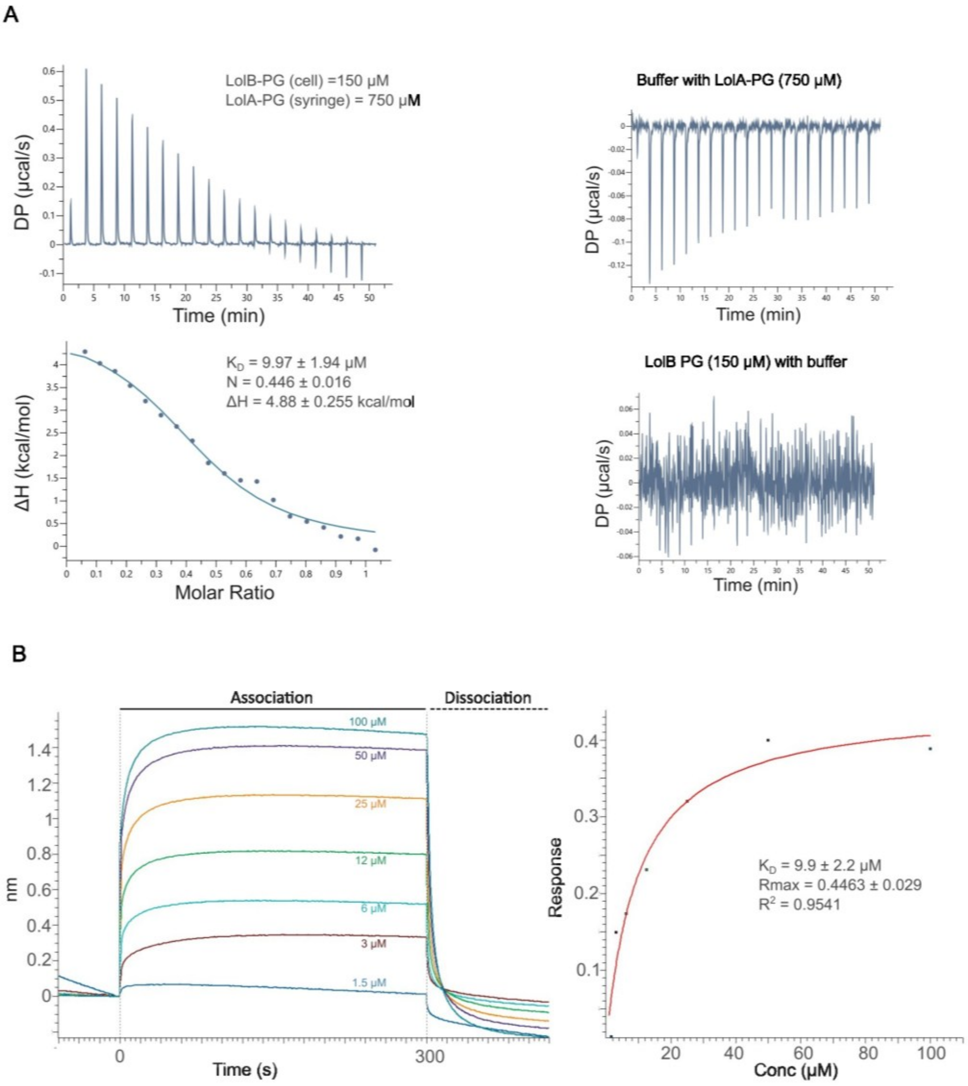

Supplementary figure S2. Interaction between LolB-PG and LolA-PG. A) ITC thermogram for LolB-PG titrated with LolA-PG (left panels). Experimental values were subtracted from heat of dilution of buffer with LolA-PG and LolB-PG (right panels). B) BLI sensogram showing raw curves (left panel) of association and dissociation traces of LolA-PG (1.5-100 µM) with His-tagged LolB-PG (1 µM) bound to Octet® NTA biosensors. The affinity plots of response vs LolA-PG concentration derived from curve fittings are shown to the right. Average values of affinities and thermodynamic parameters of is given in Table 1.

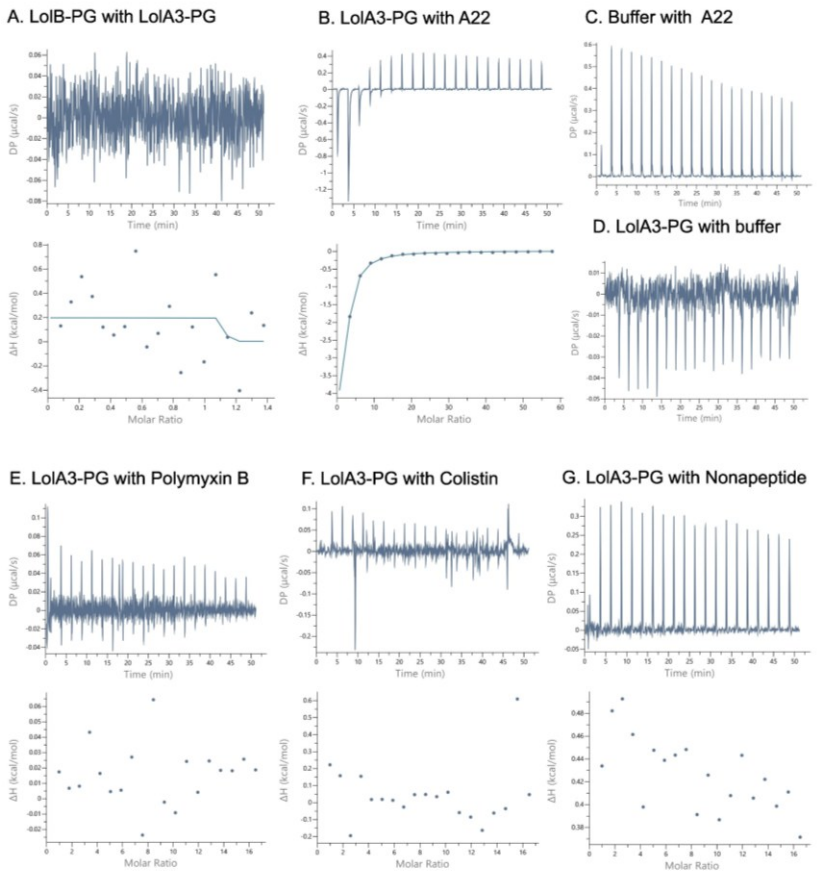

Supplementary figure S3. Thermograms for LolA3-PG. LolA3-PG is titrated with A) LolB-PG, B) A22, E) Polymyxin B, F) Colistin and G) Nonapeptide. LolA3-PG showed reproducible binding only with A22 (B). In its thermogram the heat of dilution of A22 on buffer (C) has been subtracted. Heat of dilution of 25 µM LolA3-PG (cell) with buffer is shown in D. LolA3-PG showed weak interaction with LolB-PG as shown in A. Average values of affinities and thermodynamic parameters of each repeat is given in Table 1 and 2.

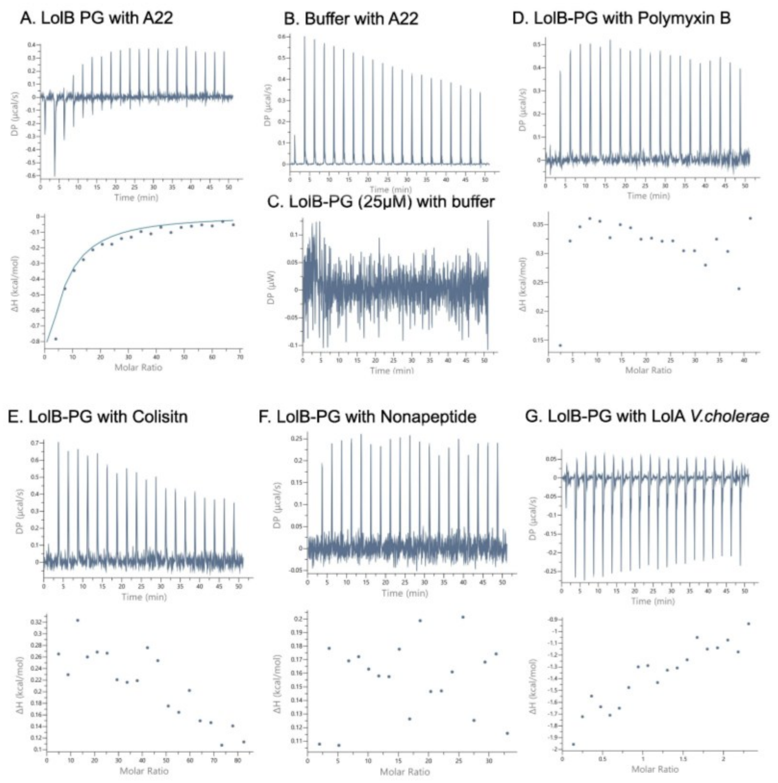

Supplementary figure S4. A representative thermogram for LolB-PG with various binding partners. A) LolB-PG is titrated with A22 which showed reproducible binding. Buffer titrated with A22 is presented in B and LolB-PG with buffer in C. LolB-PG titrated with Polymyxin B is presented in D, with Colisitin in E and Nonapeptide in F. LolB-PG was also titrated with LolA from *V. cholerae* (G) showing no interaction between the two proteins. Average values of affinities and thermodynamic parameters of each repeat are given in Table 1 and 2.

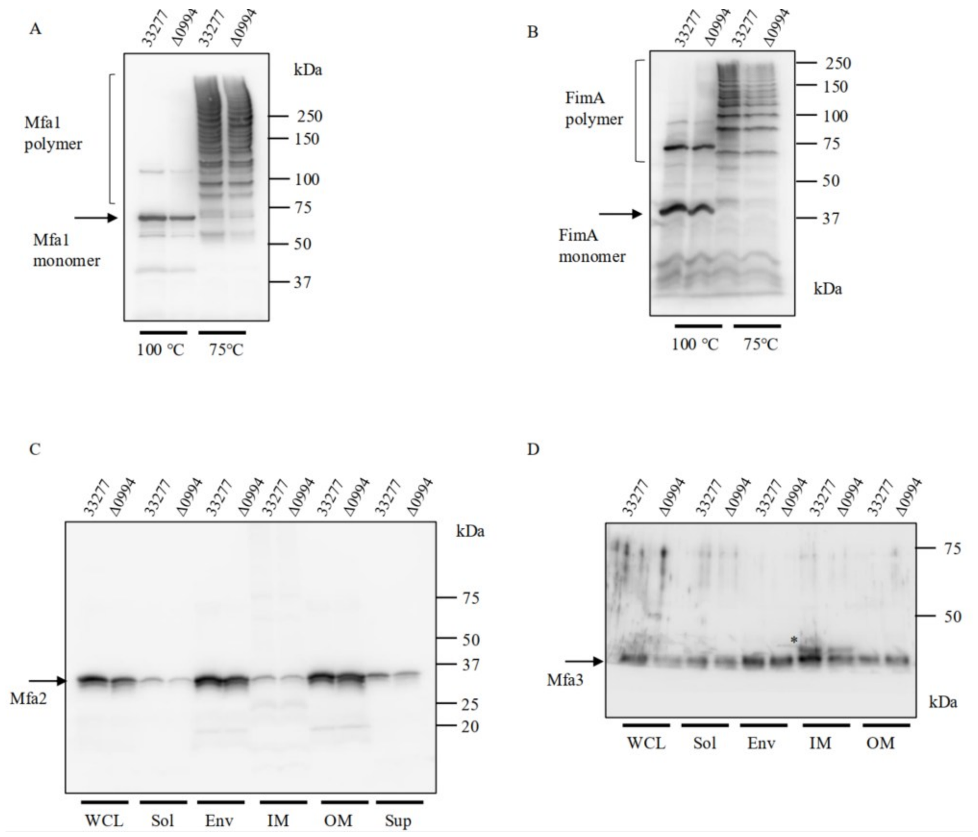

Supplementary figure S5: Comparison of fimbrial protein polymerization, localization, and maturation in wild-type *P. gingivalis* 33277 and Δ*pgn0994* by immunoblotting. A) Detection of Mfa1 polymerization. Whole-cell lysates were analyzed under two denaturation conditions: 100 °C, which fully denatures the protein into monomers, and 75 °C, which induces partial dissociation of polymers. Samples were subjected to SDS-PAGE and transferred onto PVDF membranes. Monomers and partially dissociated polymers were detected using anti-Mfa1fimbrial antibodies. B) Analysis of FimA polymerization as described in B. Monomers and polymers were detected with anti-FimA fimbrial antibodies. C) Analysis of Mfa2 localization using fractionated protein samples. Each fraction was heated at 100 °C, followed by SDS-PAGE and transfer onto a PVDF membrane. Mfa2 was detected with anti-Mfa2 antibodies. D) Analysis of Mfa3 localization and maturation. The samples were treated as in C and detected with anti-Mfa3 antibodies. Both the immature (asterisk) and mature forms of Mfa3 were detected in the IM whereas mainly the mature form was found in the OM, both in the wild-type

and mutant strain. WCL, whole-cell lysate; Sol, soluble fraction; Env, envelope fraction; IM, inner membrane; OM, outer membrane; Sup, culture supernatant.

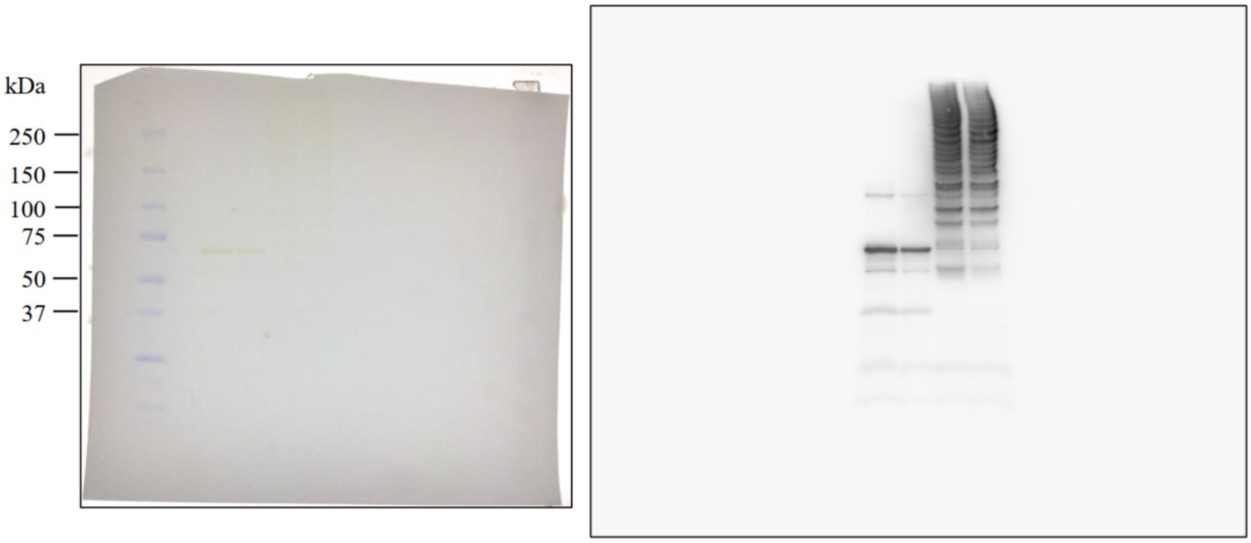

Supplementary figure S6. Uncropped immunoblot with molecular weight markers. The left panel shows the PVDF membrane after transfer, indicating the positions of molecular weight markers. The right panel shows the corresponding immunoblot. The images represent the original, uncropped blot used for supplementary figure S5A.

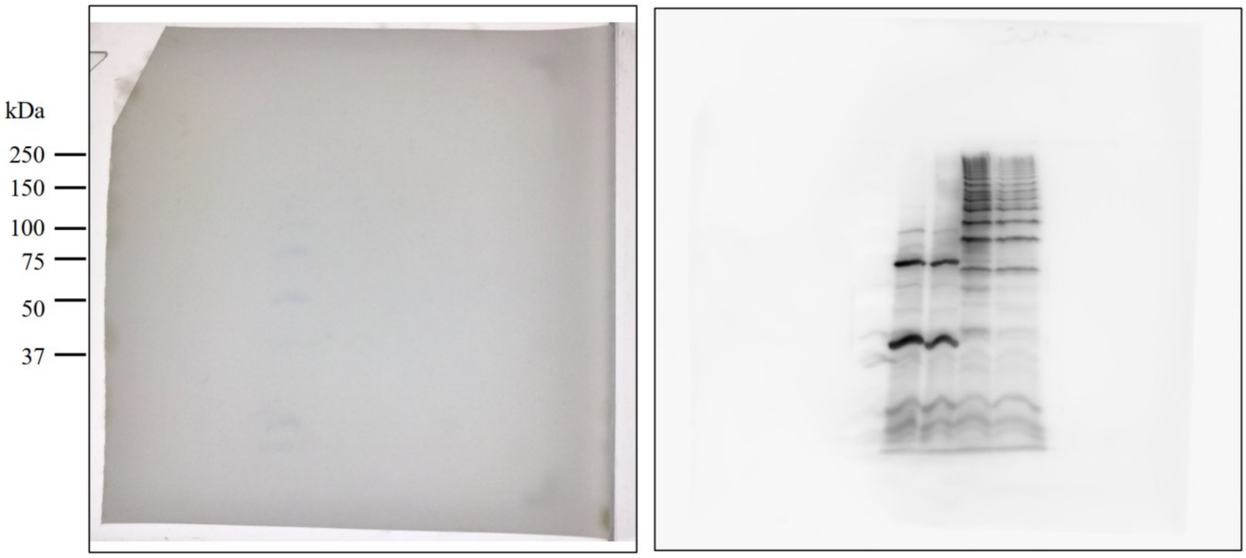

Supplementary figure S7. Uncropped immunoblot with molecular weight markers. The left panel shows the PVDF membrane after transfer, indicating the positions of the molecular weight markers. The right panel shows the corresponding immunoblot. The images represent the original uncropped blot used for supplementary figure S5B.

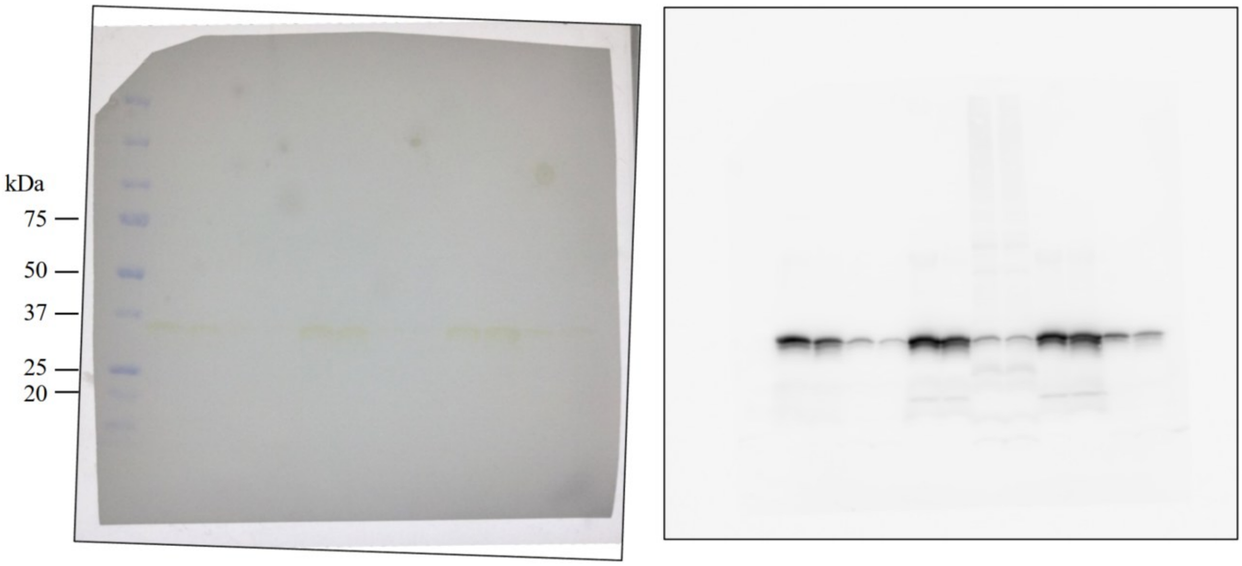

Supplementary figure S8. Uncropped immunoblot with molecular weight markers. The left panel shows the PVDF membrane after transfer, indicating the positions of the molecular weight markers. The right panel shows the corresponding immunoblot. The images represent the original uncropped blot used for supplementary figure S5C.

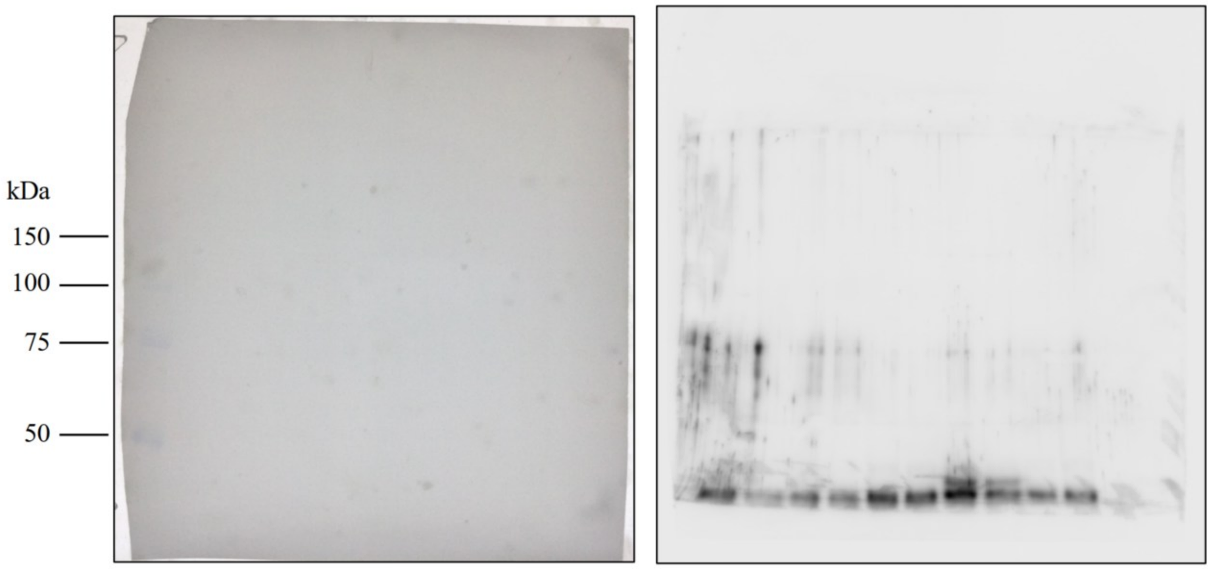

Supplementary figure S9. Uncropped immunoblot with molecular weight markers. The left panel shows the PVDF membrane after transfer, indicating the positions of the molecular weight markers. The right panel shows the corresponding immunoblot. The images represent the original uncropped blot used for supplementary figure S5D.

References:

1. Nishiyama, S.I., Hasegawa, Y. & Nagano, K. Site-Directed and Random Mutagenesis in Porphyromonas gingivalis: Construction of Fimbriae-Related-Gene Mutant. *Methods Mol Biol* **2210**, 3-14 (2021).
